# Carbon nanotube-based flexible electrothermal film heaters with a high heating rate

**DOI:** 10.1098/rsos.172072

**Published:** 2018-06-06

**Authors:** Song-Lin Jia, Hong-Zhang Geng, Luda Wang, Ying Tian, Chun-Xia Xu, Pei-Pei Shi, Ze-Zeng Gu, Xue-Shuang Yuan, Li-Chao Jing, Zhi-Ying Guo, Jing Kong

**Affiliations:** 1 State Key Laboratory of Separation Membranes and Membrane Processes, Tianjin Key Laboratory of Advanced Fibers and Energy Storage, School of Material Science and Engineering, Tianjin Polytechnic University, Tianjin 300387, People's Republic of China; 2 Department of Electrical Engineering and Computer Science, Massachusetts Institute of Technology, Cambridge, MA 02139, USA; 3 Department of Mechanical Engineering, Massachusetts Institute of Technology, Cambridge, MA 02139, USA

**Keywords:** carbon nanomaterials, transparent conducting films, film heaters, electrothermal performance, heating rate

## Abstract

High-performance, flexible film heaters with carbon nanotube transparent conducting films are easily fabricated by both a rod-coating method and a spraying method. The main conclusion we have reached is that the film demonstrates a heating rate of 6.1°C s^−1^ at 35 V and sheet resistance as low as 94.7 Ω sq^−1^ with 72.04% optical transmittance at a wavelength of 550 nm by the spraying method after a series of post-treatment processes with acid and distilled water. Then, we adopt a mathematical method of nonlinear fitting to simulate the collected experimental data and the functions effectively. Furthermore, through analysis of the formula, the correlation between temperature and time is well explained. Therefore, carbon nanotube-based, flexible, transparent heaters exhibit high electrothermal performance and are expected to find different applications, e.g. various functional devices such as heating materials, heatable smart windows or dining tables.

## 1. Introduction

With exceptional properties [1], carbon nanotubes (CNTs) attract a wide range of applications, including transparent conducting films (TCFs) [2], organic light-emitting diodes [3], composites [4], energy-related systems [5] and sensors [6]. The excellent thermal conductivity of CNT films provides a fast heating rate and a homogeneous temperature distribution with excellent optical transparency [7–9]. Electrothermal heating films, particularly transparent and flexible film heaters (FHs), have attracted attention as automobile mirrors, vehicle window defrosters, refrigerator windows, outdoor panel displays and other heating systems [10–14]. Indium tin oxide (ITO) is widely used to prepare transparent heating films because it is optically transparent to visible wavelengths and has high electrical conductivity [15,16]. However, the relatively high cost and frangibility of ITO limits it to certain applications [17,18]. In addition, a number of different of nanomaterials, such as CNTs [19] and graphene [20], are flexible and low cost, which makes them better materials for flexible electronics.

Flexible transparent heaters are fabricated from nanomaterials. CNTs have the potential to be used in electrothermal films due to their excellent thermal [21], electrical and optical properties [19]. Transparent heating films have been fabricated using single-walled CNTs (SWCNTs) with excellent heating performance. CNT films have also been shown to be excellent potential heating elements and have been investigated widely [22–24]. Compared with the materials used in the above reports, the substrate of the conductive film is different: for example, we have used a flexible poly(ethylene terephthalate) (PET) substrate that can be used not only in a plane heating environment, but also use in an irregular heating environment. However, there is trade-off between transparency and electrical resistance [25]. Wu & Wang [26] reported that the use of a double-walled CNT film heater would reduce energy consumption by up to 20–30% compared with a metal-based film heater. Jang *et al*. [27] reported that single and double sheets of multi-walled CNTs provided sheet resistances of approximately 699 Ω sq^−1^ and approximately 349 Ω sq^−1^ with transmittances of 81–85% and 67–72% at a wavelength of 550 nm, respectively. Some progress has been made, yet there is still room to improve the performance of the thin-film heater, e.g. the surface resistance and transmittance.

In this work, we carried out a series of experiments to further improve the performance of thin FHs. The electrothermal heating film-based CNTs were fabricated on flexible PET substrates using a simple solution process with a spray-coating method. We demonstrate that these heating films have a high performance with over 80% transmittance at 550 nm and reach a steady-state temperature of up to 75.5°C at an applied voltage of 30 V. Specifically, the film can be used as a heater to provide heat to maintain water at a certain temperature, such as a warm dining table to keep dishes at a suitable temperature (usually 50–60°C) for eating. The statistics were fitted by a nonlinear relation [28] to obtain a fitting formula in order to have an accurate understanding of the thermal properties of thin films [29,30]. Additionally, these films are mechanically robust, wherein repeated bending had only a slight influence on the film's thermal performance and sheet resistance. These FHs exhibit high electrothermal performance and are expected to find different applications, e.g. various functional devices such as heating materials, heatable smart windows or dining tables.

## 2. Material and methods

### 2.1. Preparation of SWCNT solutions

Ten millilitres of deionized water was slowly added to a mixture of 10 mg of SWCNTs (purity 95%, length 5–30 µm, diameter 1–2 nm) and 100 mg of sodium dodecyl benzene sulfonate (SDBS). The mixture was fabricated by bath ultrasonication exfoliation for 30 min and sonicated with a probe ultrasonicator at 120 W for 70 min. The mixture was centrifuged at 8000 r.p.m. for 20 min and the 85% supernatant was collected [31]. Finally, 100 ml of deionized water was added to the resulting suspension.

### 2.2. Fabrication of SWCNT TCFs

The PET substrates were firstly immersed in deionized water with bath ultrasonication exfoliation for 30 min to wash off the impurities. Then deionized water was replaced by ethanol to repeat the above operation in order to further remove organic impurities. The above pretreated PET substrates were prepared for later procedures. Firstly, a PET film was fixed on the heating plate at 105°C; following this, the gas flow rate of an airbrush should ensure a stable nitrogen gas flow. Finally, the spraying times were controlled to obtain TCFs with different transmittance and sheet resistance [32,33]. In this experiment, the techniques used included the spraying method, the four probe method used to measure the resistance and the flat panel display optical characteristic automatic measurement method. Owing to the influence of uncertainties in the measurements, we used the average value to reduce the error.

### 2.3. Post-treatment of TCFs

The TCFs were immersed in deionized water for 20 min, then dried at 80°C to enhance the adhesion between the SWCNTs and substrates. In order to remove the residual SDBS on and between the SWCNTs, the TCFs were rinsed in deionized water three times and dried at 80°C (water-washed TCFs). Then the water-washed TCFs were immersed in 12 M nitric acid for 30 min post-treatment [34] (acid-washed TCFs). Afterwards, the residual acid was washed away with deionized water several times and the TCFs were dried again at 80°C.

## 3. Results and discussion

**Figure 1.**
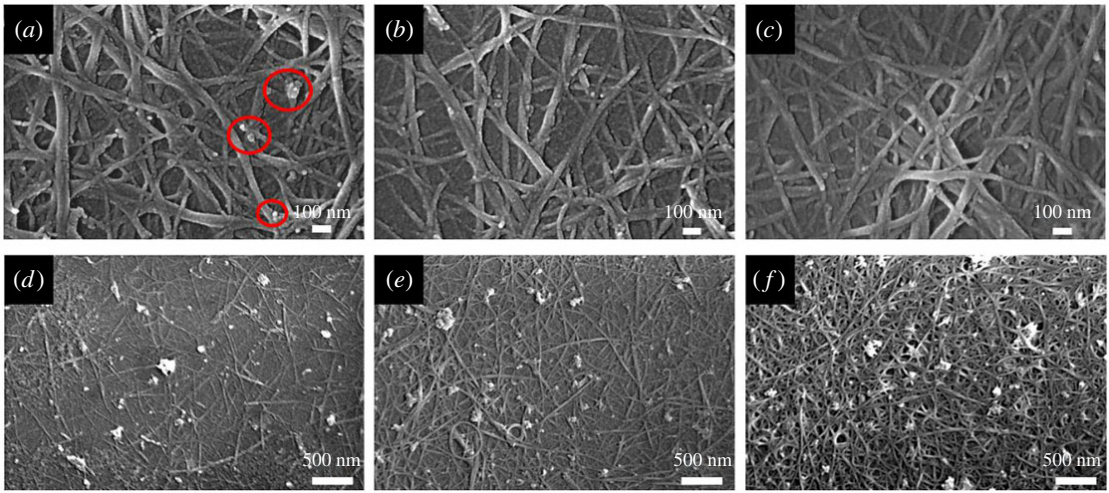
Scanning electron microscope images of (*a*) water-washed TCFs (impurities on the surface of the film are indicated by the red circles), (*b*) acid-treated TCFs, (*c*) TCFs following successive treatments with water and acid and (*d*–*f*) TCFs with a transmittance of 92.29 T %, 86.92 T % and 72.82 T % at 550 nm, respectively.

The distribution states of the SWCNT networks by different post-processing methods are compared in figure 1. The surface of the water-washed TCFs was covered with pieces of agglomerated and particulate impurities, as indicated by the red circles in figure 1*a*. By contrast, the impurities on the surface of the acid-treated TCFs were reduced, and there were only a few granular impurities among the CNT networks, as shown in figure 1*b*. Figure 1*c* shows little impurity on the surface of the TCFs that were washed with deionized water and nitric acid sequentially. The electrical conductivity changed after the nitric acid treatment, with the minimum sheet resistance decreasing to 70.47 Ω sq^−1^ (77.1 T% at 550 nm). Figure 1*d*–*f* shows various films with different transmittance (92.29 T %, 86.92 T % and 72.82 T% at 550 nm) in the visible range. With the decrease in transmittance, the distribution of CNTs on the surface became more dense so the SWCNT entangled branches link much more closely to each other in favour of increasing the transmission efficiency of electrons in the CNT networks [35].

**Figure 2.**
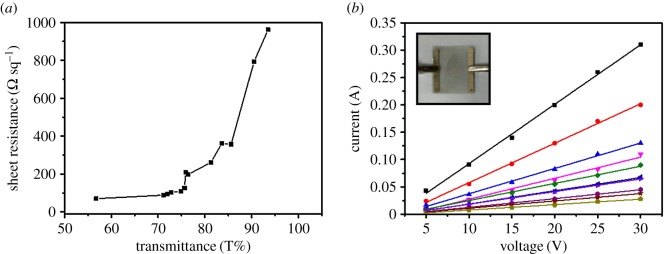
(*a*) Sheet resistance as a function of transmittance at 550 nm. (*b*) Current as a function of applied voltage measured from TCFs with different surface resistivity.

The surface resistance and transmittance of a series of CNT films were studied. Figure 2*a* shows the relationship between the surface resistance and transmittance at 550 nm. At a transmittance of approximately 85%, the surface resistance is approximately 400 Ω sq^−1^. Furthermore, the surface resistance of TCFs can be less than approximately 100 Ω sq^−1^ when the transmittance is below 75%. The reason for this is that the main factor influencing TCFs as film heaters is related to the surface resistance and does not depend on high transmittance. Therefore, a set of samples with different sheet resistance were selected for systematic investigation of the current–voltage behaviour of TCFs as film heaters, as shown in figure 2*b*.

**Figure 3.**
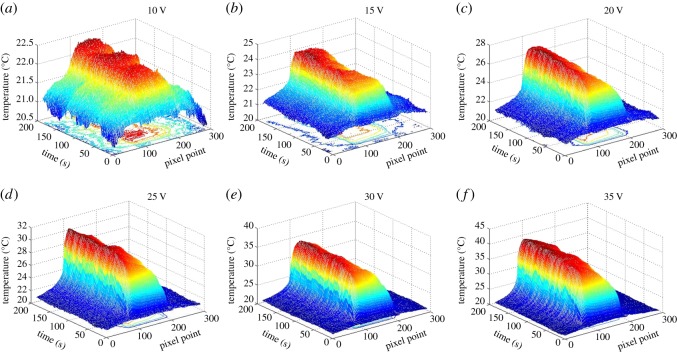
The three-dimensional coordinate images of water-treated thin FHs (3.53 kΩ sq^−1^ at 77.9 T%) reflecting the heating process with applied voltages of (*a*) 10 V, (*b*) 15 V, (*c*) 20 V, (*d*) 25 V, (*e*) 30 V and (*f*) 35 V.

**Figure 4.**
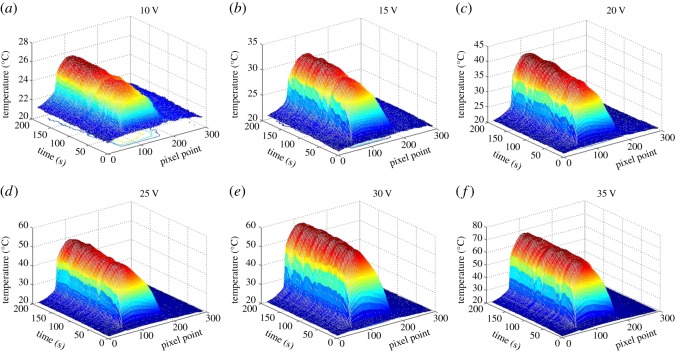
The three-dimensional coordinate images of acid-treated thin FHs (0.66 kΩ sq^−1^ at 81.1 T %) reflecting the heating process with applied voltages of (*a*) 10 V, (*b*) 15 V, (*c*) 20 V, (*d*) 25 V, (*e*) 30 V and (*f*) 35 V.

The three-dimensional thermal infrared images of the TCFs with two different post-processing methods are shown in figure 3 and figure 4, and were measured when the voltage was increased from 10 V to 35 V. The *x*-axis represents time up to 200 s for the whole process. The *y*-axis shows the number of pixels, up to 300, representing the position along the wide direction of the sample. The *z*-axis shows the temperature, which increases rapidly with time to a steady state. The steady-state temperature of the film heater increases with the decrease in the film surface resistance. In figure 3*a*, this could be due to the lower voltage as the temperature fluctuates along the film surface because of the small temperature difference from the environmental temperature at a lower voltage. However, the steady­-state temperature fluctuated slowly with the input voltage, increasing from approximately 22°C to approximately 38°C. Compared with water-treated TCFs, the fluctuation of acid-treated TCFs is much less and the steady state can reach a relatively higher temperature, even up to approximately 70°C, as shown in figure 4. The three-dimensional thermal infrared images of the FHs show that FHs are very sensitive to mild temperature fluctuations and have excellent heating performance.

**Figure 5.**
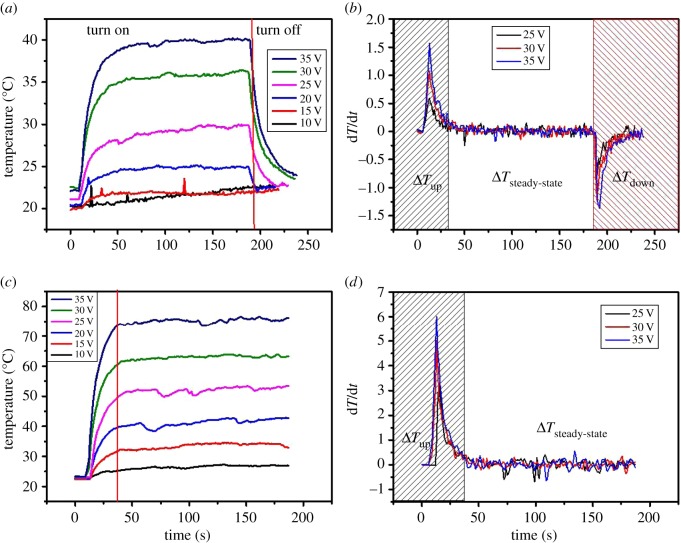
(*a*) The time-dependent temperature profiles of the water-washed TCF heaters as a function of the applied voltage. (*b*) Heating rate curves of the water-washed TCF heaters at 25 V, 30 V and 35 V. (*c*) The time-dependent temperature profiles of the acid-washed TCF heaters as a function of applied voltage. (*d*) Heating rate curves of the acid-washed TCF heaters at 25 V, 30 V and 35 V.

In order to study the electrothermal properties of the FHs by different preparation processes, a series of heating experiments were conducted and the experimental results are shown in Figure 5. Figure 5*a* shows the electrothermal performance of the water-washed TCFs under different applied DC voltages from 10 to 35 V. The optoelectronic performance of the film was 3.53 kΩ sq^−1^ with a corresponding transmittance of 77.9% at 550 nm. The steady-state temperature (*T*_steady-state_) increased from 22.3°C to 40.7°C while increasing the power voltage from 10 to 35 V. The response time [36], which is defined as the time to reach approximately 90% of the *T*_steady-state_ from room temperature [37], is also one of the key factors for evaluating the performance of FHs. It can be clearly seen that the response time of the electrothermal film is less than 30 s, illustrating its fast response characteristic. Compared with the previously reported graphene heaters' response time of about 60–150 s, the SWCNT-TCF heaters show a relatively shorter response time, possibly benefiting from the lower sheet resistance. When the power was turned off, the temperature of the electrothermal films decreased to the initial state, namely room temperature. The heating rate curves of the water-washed TCFs in figure 5*b* were obtained by calculating the first-order derivative of the time-dependent temperature curves. The fast response feature was further exhibited by the heating rate curves. Water-washed TCFs show a steady-state temperature of 40.7°C with a maximum heating rate of 1.5°C s^−1^ at a driving voltage of 35 V. Figure 5*c* shows the electrothermal performance of the acid-washed TCFs under different applied DC voltages from 10 to 35 V. The optoelectronic performance of the film was 0.66 kΩ sq^−1^ with corresponding transmittance of 81.1% at 550 nm. The electrothermal films demonstrated a steady temperature increase from 25.7°C to 75.5°C by increasing the voltage from 5 to 30 V. As shown in figure 5*d*, the heating rate curve of the acid-washed TCFs has a maximum heating rate of 6.1°C s^−1^ at 35 V. This value is closely related to the size of the film (the film size is 5 × 5 cm in this experiment). According to these experimental results, acid-washed FHs exhibited the better performance including higher steady-state temperature and higher heating rate. The better performance of acid-washed FHs is the result of lower sheet resistance because of immersing the FHs in nitric acid to remove the residual SDBS on and between the SWCNT networks, and this makes the SWCNTs connect to each other well to form conductive paths.

**Figure 6.**
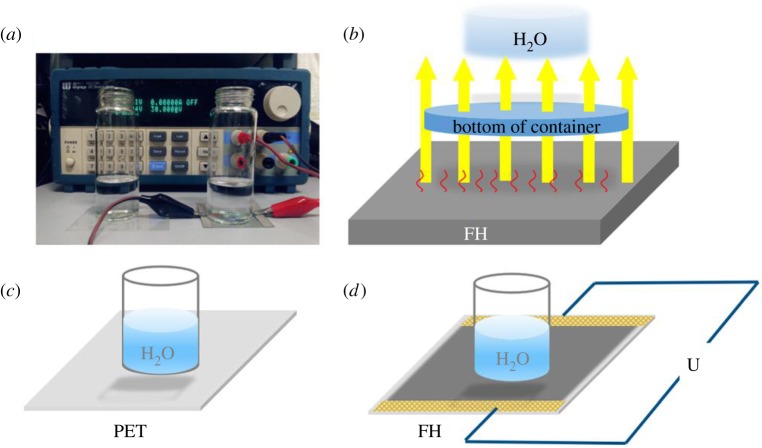
(*a*) Device for the measurement of heating characteristics of SWCNT-TCF heaters. (*b*) Schematic illustration of the heat transfer process. Schematic illustration of (*c*) pure PET substrate and (*d*) SWCNT-TCF heaters placed in a glass of water.

Figure 6*a* shows the device for the measurement of heating characteristics of SWCNT-TCF heaters. The instrument (Maynuo DC Source Meter-M8811) provides a stable voltage for the film heater to allow the heater to generate heat. A schematic illustration of the heat transfer process is shown in figure 6*b*. The schematic illustrations in figure 6*c*,*d* compare pure PET substrate with SWCNT-TCF heaters placed in a glass of water.

**Figure 7.**
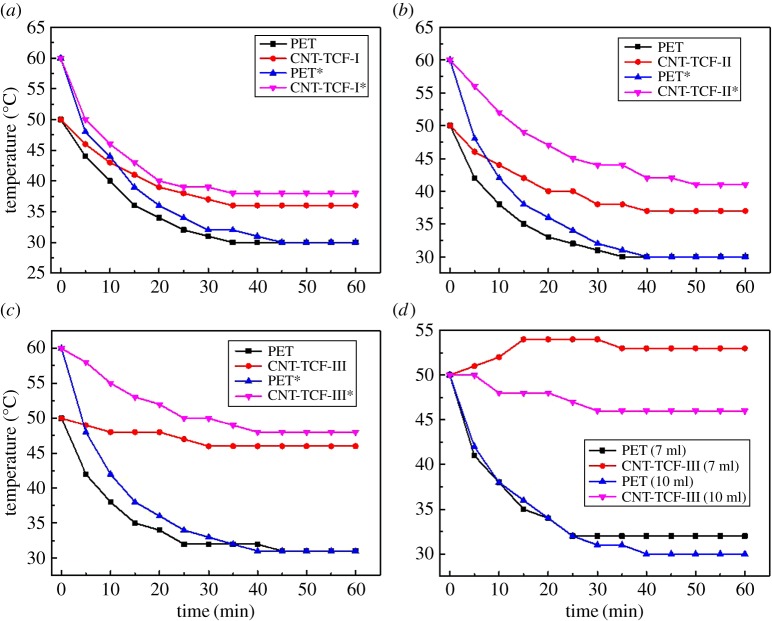
The cooling curves of the water with different initial temperature of (*a*) CNT-TCF-I (357.08Ω sq^−1^ at 85.63 T%), (*b*) CNT-TCF-II (208.4 Ω sq^−1^ at 75.9 T%), (*c*) CNT-TCF-III (94.7 Ω sq^−1^ at 72.04 T%). (*d*) The cooling curve of the water with different volumes of water with CNT-TCF-III.

For the application of FHs in a dining table, a safe voltage of 36 V is usually needed to maintain a temperature of around 50–60°C. The thermal properties of the CNT-TCFs fabricated by the spraying method were further studied. The films were applied at a voltage of 30 V to obtain the cooling curves by measuring the temperature of the deionized water. The results are illustrated in figure 7. As shown in figure 7*a*–*c*, they show the trend of the water temperature of the PET and CNT-TCF under different initial temperatures beginning with an initial room temperature of 30°C. Considering the actual application, such as heating devices or defrosters, 50°C and 60°C were used as the initial temperatures in this work, respectively, with PET as a blank control experiment. In figure 7*a*, the red line and black line represent the CNT-TCF-I substrate and PET substrate with an initial temperature of 50°C, respectively. It can be clearly seen that the temperature difference is about 6°C and the water temperature is almost a constant within 60 min. The steady-state temperature difference is approximately 8°C when the initial temperature is 60°C. Compared with CNT-TCF-II and CNT-TCF-III, CNT-TCF-I has a relatively higher sheet resistance and displays the lowest heat conversion efficiency at the same applied voltage. Therefore, CNT-TCF-I can be used as a transparent heating film on either cars or building windows to remove frost because of its low electricity consumption and good transparency of higher than 85% over the whole visible region. Furthermore, as shown in figure 7*b*,*c*, a higher steady-state temperature difference is observed for CNT-TCF-II and CNT-TCF-III, which benefit from lower sheet resistance. For CNT-TCF-II, the water temperature reaches a steady state of 37°C higher than the room temperature of 30°C when the initial temperature was 50°C and the steady-state temperature difference is about 12°C with an initial temperature of 60°C. With regard to CNT-TCF-III, the time taken to reach the steady-state temperature is less than for the others and the final temperature can reach 46°C and 48°C at an initial temperature of 50°C and 60°C, respectively. We also explore the heating properties of the CNT-TCF with different volumes of liquid in figure 7*d*. It can be seen that the cooling curves (red line) rise slightly at 15 min and then achieve a balanced state when the volume of the water is 7 ml. The steady-state temperature is 53°C after 35 min. Therefore, the volume of the liquid also affects the final steady-state temperature. Compared with CNT-TCF-I, CNT-TCF-II and CNT-TCF-III samples can achieve a higher input power and steady-state temperature of water at the same applied voltage, especially CNT-TCF-III because of the relatively low sheet resistance.

To explore the heating properties and has heat-insulating properties of thin films, the thermal properties of the films were analysed from the point of view of the heat quantity, and the schematic diagram of the heat transfer process as shown in figure 6*b*. The quantity of heat absorption or heat dissipation can be estimated from

**Figure 8.**
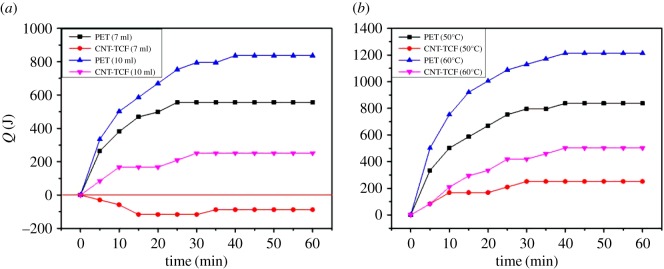
(*a*) The heat released by different volumes of hot water as a function of time with PET and CNT-TCF-III as the substrate, respectively. (*b*) The heat released by hot water as a function of time with PET and CNT-TCF-III as the substrate at initial temperatures of 50°C and 60°C, respectively.

3.1 Q=c×m×ΔT,

where *c* represents specific heat capacity (the value for glass is 840 J/(kg °C) at 25°C, the value for ceramics is 837 J/(kg °C) at 25°C) and *m* is the mass of substance [38,39]. Δ*T* represents the temperature difference between the starting temperature and the equilibrium temperature. *Q* is plotted to analyse the variation in the rules of heat transfer with water of different volumes at an initial temperature of 50°C in figure 8*a*. The quantity of heat released by water increases continuously with time when the volume of water is 10 ml, reaching an equilibrium position after 30 min. The final release of heat based on CNT-TCF-III is 251.2 J and the value with pure PET is 837.2 J. When the volume of water was 7 ml, the water with PET substrate release of heat is 556.7 J ultimately, while the water with CNT-TCF-III as the substrate not only had no heat release, but also absorbed heat from the substrate of about 87.9 J. Figure 8*b* shows that the heat released by hot water increases with time when the volume of water is 10 ml at different initial temperatures. It is observed that the heat of the water based on PET substrates reduces by more than 800 J at an initial temperature of 50°C, even reaching 1213.9 J at 60°C. By contrast, the water with CNT-TCF-III substrate produces heat of about 502.3 J at an initial temperature of 60°C, and produces even less heat at 50°C. Therefore, the CNT-TCFs have better thermal performance and are suited to more irregular devices because of their flexibility.

To understand the relationship between temperature and time quantitatively, the cooling curves of the water temperature that dropped from an initial temperature to room temperature were fitted using the exponential function

**Figure 9.**
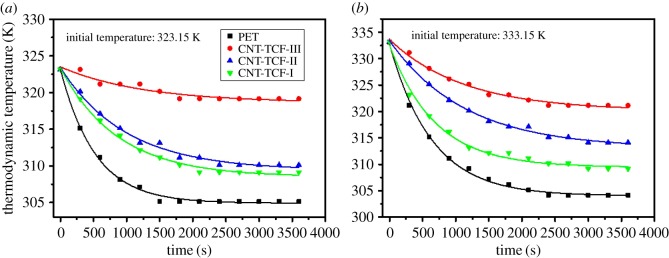
The fitted cooling curves of water at initial temperatures of (*a*) 323.15 K and (*b*) 333.15 K.

3.2 $$T=T^{*}+T_{c}\times e^{-t/\lambda },$$

where T*, T_c_ and *λ* are unknown parameters and can be solved through nonlinear fitting, *T* and *t* are real-time temperature and time, respectively. In figure 9*a*,*b*, the black square represents water temperatures with only PET when the room temperature is 303.15 K, defined as *T_R_*, in the whole process. When the initial temperature *T_i_ *= 323.15 K, by fitting the PET curve in figure 9*a*, we can obtain the parameters as follows: T* = 304.92, *T_c_ *= 18.27 and *λ* = 526.5. The value of T* (=304.92) should actually be the ambient temperature *T_R_*. *T* will be 323.19 with *t* = 0, which should be equal to the initial temperature *T_i_* = 323.15 K. The value of *T_c_* (=18.27) is the maximum temperature difference between the hot water and the environment, that is, approximately equal to the value of (*T_i_* − *T_R_*). The calibration factor *λ* (=526.5) is used to calibrate the real-time temperature at an initial temperature of 323.15 K. By fitting the PET curve in figure 9*b*, we obtained the parameters as follows: T* = 304.02, *T_c_ *= 28.71 and *λ* = 642.3, owing to a different initial temperature *T_i_* = 333.15 K. According to nonlinear fitting, we summarize the general formulae and the specific parameters as shown in table 1.

**Table 1.** The parameters of the formula obtained by curve fitting of figure 9.

initial temperature (323.15 K)	T*	*T_c_*	*λ*	initial temperature (333.15 K)	T*	*T_c_*	*λ*
CNT-TCF-III (94.7 Ω sq^−1^)	318.62	4.89	1247.8	CNT-TCF-III (94.7 Ω sq^−1^)	320.15	13.46	1130.4
CNT-TCF-II (208.4 Ω sq^−1^)	309.42	14.00	989.8	CNT-TCF-II (208.4 Ω sq^−1^)	313.05	20.35	1021.3
CNT-TCF-I (357.08 Ω sq^−1^)	308.51	14.84	868.7	CNT-TCF-I (357.08 Ω sq^−1^)	309.46	23.10	687.3
pure PET substrate	304.92	18.27	526.5	pure PET substrate	304.02	28.71	642.3

Based on the above equations, we find that the T* is increasing with the decrease in the sheet resistance from CNT-TCF-I to CNT-TCF-III. In this situation, the value for T* can be defined as the steady-state temperature when the hot water temperature is a constant during 60 min, meanwhile it can reflect the heating performance of the flexible thin film intuitively. In terms of the fitting equation, the maximum temperature difference between the hot water and the surroundings is *T_c_*, which decreases with a reduction in sheet resistance. The heat conduction performance of the film is reflected by the value *T_c_*, whose value is related to the film itself, and each film only corresponds to a fitting value. The surface resistance of the thin-film heater plays a significant role when the voltage *U* is supplied, the power generated by the film heater is *p = U ^2^/ Rs*, which is inversely proportional to *R_s_*. Therefore, according to the fitting parameters, we deduced that the parameters in the fitting formula can be expressed as follows:

3.3 $$\begin{array}{rl} T^{*} & \;=T_{R}\left(1+\frac{\alpha }{R_{s}}\right), \end{array}$$

3.4 $$\begin{array}{rl} T_{c} & \;=(T_{i}-T_{R})\left(1-\frac{\beta }{R_{s}}\right) \end{array}$$

3.5 $$\begin{array}{rl} \text{and}\;\lambda & \;=\lambda_{0}\left(1+\frac{\gamma }{R_{s}}\right), \end{array}$$

where *α*, *β*, *γ* and *λ_0_* are the correction parameters related to the film parameters, applied voltage, initial temperature and the environmental conditions, respectively.

From the above descriptions, we have demonstrated a more detailed research into the heating performance of thin films, and qualitatively analysed the relationship between temperature change and time. This not only provides a possibility for a more complicated heating environment, but also qualitatively controls the change in temperature. The real-time temperature can be calculated by the formulae of nonlinear fitting about thin FHs with different surface resistance based on CNTs. Thin-film heaters are promising to replace traditional heaters in areas such as warm dining tables, smart windows and more intelligent hardware due to these merits of smaller volume, flexibility, transparency, faster heating rate and adjustable temperature by changing the sheet resistance and applied voltage.

## 4. Conclusion

In this work, we have fabricated SWCNT-based flexible transparent film heaters with excellent heating performance through spray coating on a PET substrate. Because of the existence of random errors and the influence of the surrounding environment, we used multiple measurements to obtain the average. Through the three-dimensional thermal infrared images, we could intuitionally observe the thermal distribution and the temperature rising trend of the FHs. Then the rate curve can be observed very intuitively, the increasing temperature curve exhibited a slower response time of about 30 s and the heating rate was up to 6.1°C s-1 at 35 V. After that, the heating performance of FHs were measured through heating water with a certain initial temperature compared with pure PET substrate and equations were solved via nonlinear fitting, with the aim of obtaining more accurate real-time temperatures. Hence, the FHs have the advantages of chemical stability, mechanical flexibility, high transparency over 80% transmittance at 550 nm, light weight due to the PET substrate, and scalable production through easy solution processing. The FHs exhibit a fast heating response of less than 30 s, homogeneous steady temperature distribution in which the average fluctuates by less than 1.5°C as well as excellent flexibility. The high heating rate of FHs fabricated by SWCNT TCFs could be a good choice for wide applications of flexible heating devices.

## Data accessibility

This article does not contain any additional data.
